# Prognostic significance of Daxx NCR (Nuclear/Cytoplasmic Ratio) in gastric cancer

**DOI:** 10.1002/cam4.1144

**Published:** 2017-08-15

**Authors:** Jian‐feng Xu, Zhi‐guang Zhao, Le‐le Ye, Weishan Zhuge, Zheng Han, Te‐ming Zhang, Si‐si Ye, Wen‐jing Chen, Shanli Zhu, Li Shi, Jun Zhang, Ai‐zhen Guo, Xiang‐yang Xue, Xian Shen

**Affiliations:** ^1^ Department of Gastrointestinal Surgery The First Affiliated Hospital Wenzhou Medical University Wenzhou Zhejiang China; ^2^ Department of Pathology The Second Affiliated Hospital Wenzhou Medical University Wenzhou Zhejiang China; ^3^ Basic Medical College of Wenzhou Medical University Wenzhou Zhejiang China; ^4^ Department of Gastrointestinal Surgery The Second Affiliated Hospital Wenzhou Medical University Wenzhou Zhejiang China; ^5^ Department of Gastroenterology Jinhua First People ‘s Hospital Jinhua Zhejiang China; ^6^ Division of Hematology, Oncology and Blood & Marrow Transplantation Department of Internal Medicine Holden Comprehensive Cancer Center University of Iowa Carver College of Medicine Iowa City Iowa; ^7^ Department of Internal Medicine Yangpu Hosptial Tongji University School of Medicine Shanghai China

**Keywords:** Chemotherapy, Daxx, gastric cancer, subcellular localization, survival

## Abstract

In addition to regulating apoptosis via its interaction with the death domain of Fas receptor, death domain associated protein 6 (Daxx) is also known to be involved in transcriptional regulation, suggesting that the function of Daxx depends on its subcellular localization. In this study, we aimed to explore Daxx subcellular localization in gastric cancer (GC) cells and correlate the findings with clinical data in GC patients. Seventy pairs of tissue samples (GC and adjacent normal tissue) were analyzed immunohistochemically for Daxx expression and localization (nuclear and cytoplasmic). The Daxx Nuclear/Cytoplasmic ratio (Daxx NCR) values in tissue microarray data with 522 tumor samples were further analyzed. The defined Prior cohort (*n* = 277, treatment between 2006 and 2009) and Recent cohort (*n* = 245, treatment between 2010 and 2011) were then used to examine the relationship between Daxx NCR and clinical data. The Daxx NCR was found to be clinically informative and significantly higher in GC tissue. Using Daxx NCR (risk ratio = 2.0), both the Prior and Recent cohorts were divided into high‐ and low‐risk groups. Relative to the low‐risk group, the high‐risk patients had a shorter disease free survival (DFS) and overall survival (OS) in both cohorts. Importantly, postoperative chemotherapy was found having differential effect on high‐ and low‐risk patients. Such chemotherapy brought no survival benefit, (and could potentially be detrimental,) to high‐risk patients after surgery. Daxx NCR could be used as a prognosis factor in GC patients, and may help select the appropriate population to benefit from chemotherapy after surgery.

## Introduction

Gastric cancer is a gastrointestinal malignancy with one of the poorest prognoses and is currently the malignancy with the second highest mortality rate in China 1. The principal means of treatment is through surgical resection, which is widely considered as a radical form of treatment. Many patients with gastric cancer are diagnosed very late in their disease, thereby missing the optimal treatment time. Of those patients who undergo surgical resection, many eventually experience a recurrence of the cancer, with or without metastasis, after surgery; the 5‐year overall survival rate is often less than 20% 2. Accurate assessment of the prognosis, before and after treatment, is helpful in choosing the most appropriate treatment for patients with gastric carcinoma. While TNM is valuable to predict prognosis, it is limited by its lack of information at the molecular level. Therefore, an expansion of prognostic markers is urgently needed to reflect the complexity of neoplasia, as well as to provide a more accurate picture to guide our clinicians.

Daxx is a Death domain‐associated protein that binds specifically to the Death‐domain of the Fas receptor. Owing to post‐transcriptional processing, human death domain associated protein 6 (Daxx) exists in three isoforms, with molecular weights of 70, 97, and 120 kDa, respectively. Human Daxx has four domains: two double helix domains, one acidic amino acid‐rich domain, and one domain that is rich in serine/proline/threonine residues. The four domains are closely related to the regulation of Daxx transcription 3.

The earliest reports describing Daxx function showed that overexpression of Daxx enhanced Fas‐mediated apoptosis and activated the JNK pathway 4. However, there is currently no consensus on the function of Daxx: the interaction between Daxx and the Fas receptor suggests that Daxx plays a functional role in the cytoplasm, whereas other reports suggest that Daxx is mainly located in the nucleus 5, 6. Daxx may therefore have different roles in the cytoplasm and the nucleus. In fact, it has been reported that in neurons, the function of Daxx varies according to its localization: for example, when Daxx is located in the nucleus, it is antiapoptotic, whereas when it is localized in the cytoplasm, it is proapoptotic 7. Numerous studies have been conducted examining the expression of, or mutations in, Daxx in tumor tissues, such as in oral cancer, pancreatic neuroendocrine tumors, urothelial carcinoma, prostate cancer, and ovarian cancer amongst others 8, 9, 10, 11, 12. In tumor cells, Daxx is mainly located in the nucleus and is considered as a cancer‐promoting factor. For example, patients with diffuse large B‐cell lymphoma that have high Daxx expression tend to have a worse prognosis 13. In keeping with this notion, Daxx‐inactivating mutations in patients with pancreatic cancer have a better survival rate 14. Studies similar to these, however, have rarely been reported in gastric cancer, and the issue regarding subcellular localization of Daxx in gastric cancer has been largely ignored. It is well known that a protein's subcellular locations may be associated with different functions. In fact, numerous studies have shown that protein subcellular localization can be associated with functions that are closely related to tumor progression 15, 16, 17, 18, 19.

On the basis of these considerations, the aim of this study was to examine the subcellular localization of Daxx in tissue samples from gastric cancer patients in the hope that this might provide some novel insights into the treatment and prognosis of gastric cancer.

## Materials and Methods

### Study patients

We obtained pathologically proven, formalin‐fixed paraffin embedded (FFPE) specimens from 620 GC patients, including 70 GC patients with a paired adjacent normal tissue sample. All of these patients had received curative surgery in The Second Affiliated Hospital of Wenzhou Medical University (Wenzhou, China) between December 2006 and December 2011. Due to the updates in the guideline of surgical treatment, as well as improvements in surgical techniques, we decided to divide the patients into two cohorts. Of these, 334 GC patients received curative surgery from December 2006 to December 2009 and were placed in the Prior cohort, which included 233 men and 101 women with a median age of 59 years‐old and an interquartile range of 20–99 years‐old. The other 286 GC patients who received curative surgery from December 2010 to December 2011 were placed in the Recent cohort, which included 204 men and 82 women with the median age of 66 years‐old and an interquartile range of 37–90 years‐old. The clinical characteristics of each patient included the age, gender, tumor size, differentiation status, TNM stage at the time of surgery (TNM stage was classified according to the American Joint Committee on Cancer Staging Manual 7th edition), and guideline based postoperative chemotherapy. We also acquired FFPE specimens of normal gastric mucosa tissues from 23 patients who had undergone elective bariatric surgery. This study was approved by the institutional review board of the Second Affiliated Hospital. All paients were aware of the research and signed the informed consent form.

Patient follow‐up was performed though outpatient services every 21 days, or if the patient developed symptoms that required a visit to a local clinic or hospital. Serum carcinoembryonic antigen (CEA) and carbohydrate antigen 19‐9 (CA 19‐9) levels were monitored in the regular follow‐ups. The final follow‐up occurred in June 2016.

### Outline of the experimental approach

**Figure 1:**
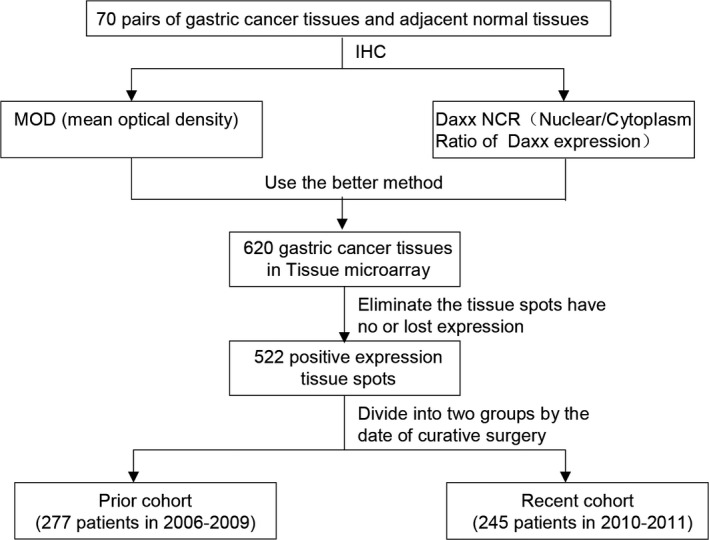
Outline of our experimental approach.

The seventy pairs of matched samples were first examined by immunohistochemistry (IHC) to confirm the location and expression of Daxx in GC tissue. The mean optical density (MOD) of nuclear Daxx expression was used as a measure of nuclear Daxx expression. As part of this study, we also calculated the nucleus/cytoplasm ratio (NCR) using the MOD of Daxx expressed in the nucleus over the cytoplasm. This was performed to determine which read‐out (the absolute MOD or NCR) was most informative, and then use this readout to analyze a tissue microarray of a larger number of GC tissue samples. An outline of our experimental approach is shown in Figure 1.

### Construction of TMA

We retrospectively collected formalin‐fixed, paraffin blocks of 620 GC patients from the Second Affiliated Hospital. Each specimen has the donor information of age, gender, disease location, TNM stage at the time of surgery, and status of postoperative adjuvant chemotherapy. Before the construction of the tissue microarray (TMA), hematoxylin and eosin (HE) stained slides from the blocks were reviewed by qualified pathologists to circle the interested region for coring. A 0.6‐mm‐diameter core in the circled region was released with a needle from each block, arrayed, and re‐embedded in a recipient block. For standardization between different recipient blocks, in‐cohort controls of the cores from eight specimens were presented on every array‐block. There were no redundant cores for each patient on the array‐block. After TMA construction, a HE section of the recipient block was reviewed to confirm that the interested region was contained in the cores. TMA cutting was performed by a skilled pathologist and all finished slides were dipped in paraffin for preservation at 4°C before immunohistochemistry assays.

### Immunohistochemistry and detection of DAXX expression

First, IHC was carried out on the 70 tissue pairs in the Pathology Laboratory of The Second Affiliated Hospital of Wenzhou Medical University, using rabbit anti‐Daxx polyclonal antibodies at 1:100 dilution ratio (#sc7152; Santa Cruz Biotech., Santa Cruz, CA) according to the manual. Positive criteria were initially confirmed by two pathology experts, and if a third expert also confirmed the result it was accepted. In the event that the third expert disagreed, the data were reviewed by all three experts and discussed until a consensus result could be reached. Immunohistochemistry was performed in a similar way for the negative control group, and the results are presented in the FigureS1. The same IHC approach was used for the detection of DAXX in tissue microarrays (TMAs).

### Detection of Daxx NCR ratio

Three microscope images (400x magnification) were obtained for each tissue sample, and every view included at least 100 Daxx‐positive cells. In the final analysis, negative spots, microarray spots with no tumor tissue, and missing spots were eliminated. Image analysis data were expressed as MOD (including both nuclear and cytoplasmic staining) and was processed using Image Pro Plus 6 (IPP; produced by Media Cybernetics Corporation, USA) to calculate the nuclear/cytoplasmic Daxx expression ratio (Daxx NCR).

### Statistical analysis

**Figure 2:**
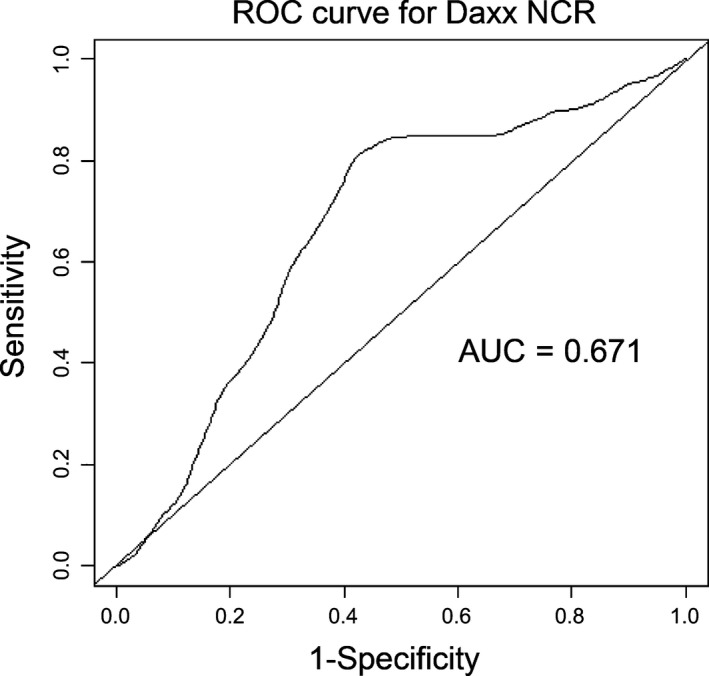
The time‐dependent receiver operating curve curves for Daxx NCR in patients with gastric cancer according to Overall Survival.

As described above, Image Pro Plus 6 was used to analyze the immuno‐histochemical images for Daxx expression. The output from this analysis was expressed as MOD. The MOD values in both the nucleus and the cytoplasm in positive tumor cells were separately recorded and then used to calculate the Daxx NCR. A chi‐squared test was used to compare Daxx expression in tumor tissue with expression in adjacent normal tissue. A paired *t*‐test was performed to compare Daxx nuclear expression, as well as the nuclear/cytoplasmic Daxx expression ratio, between tumor tissue and adjacent normal tissue. The cut‐off point, which categorized patients into high‐risk and low‐risk subgroups, was optimized using a receiver operating curve (ROC), as shown in Figure 2. The values (cutoff values = 2.0) with the maximal Youden index were chosen as the cutoff points for the Daxx NCR.

To determine differences in clinico‐pathogical variables between high‐risk and low‐risk subgroups in both cohorts, we performed a *t*‐test for the continuous variable and a chi‐square test for categorical variables. A Kaplan–Meier's analysis with a log‐rank test was performed to estimate disease‐free survival (DFS) and overall‐survival (OS). Univariate and multivariate Cox regression analysis was performed to determine the contribution of Daxx expression to patient survival, adjusting for gender, age, tumor size, tumor differentiation grade, lymph nodes examined, TNM stage, serum CEA, serum CA19‐9, and adjuvant chemotherapy. All statistical analysis was performed using R and SPSS V.23 for Windows (SPSS, Chicago, IL). Significance was defined as *P *<* *0.05.

## Results

### Differences in Daxx expression between GC tissues and adjacent normal tissues

**Figure 3:**
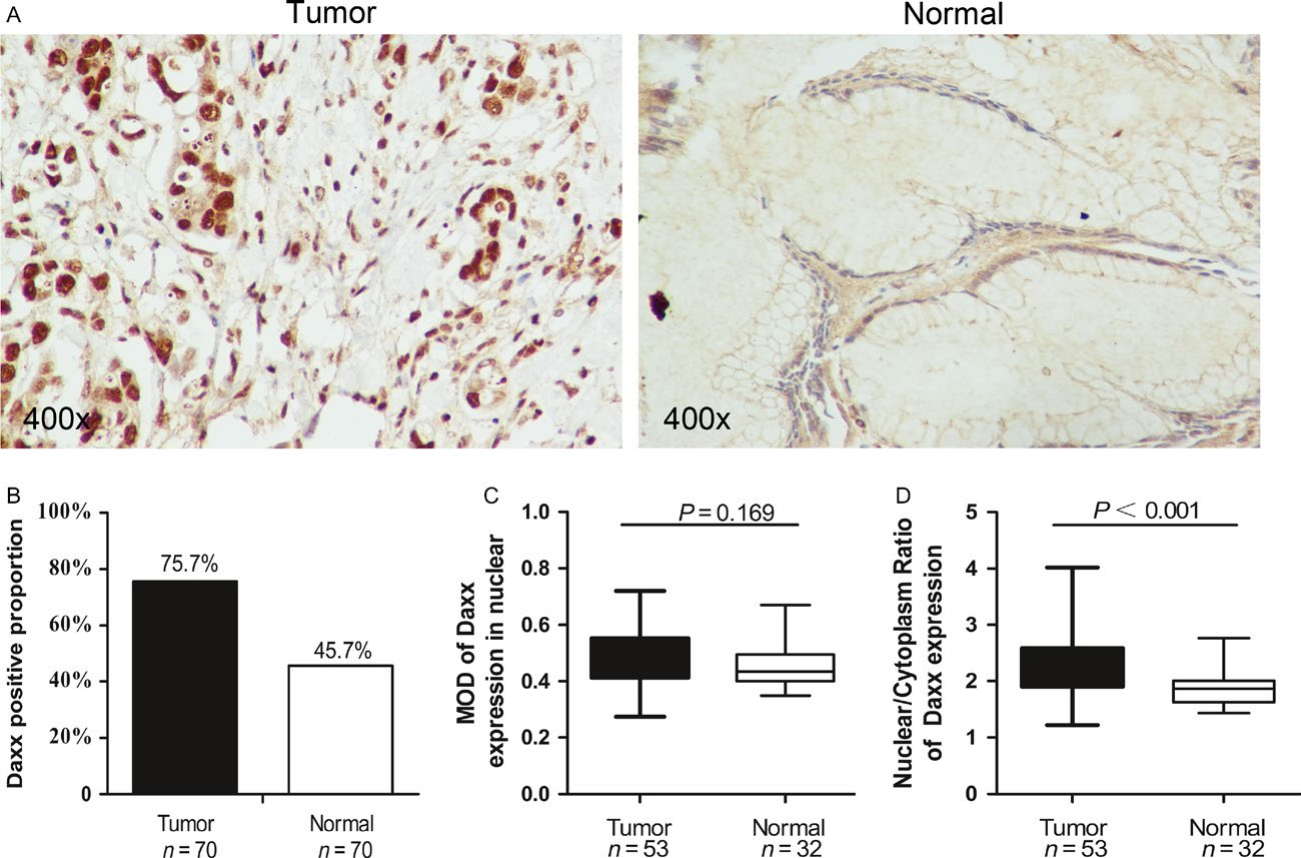
Daxx expression in gastric cancer and adjacent normal tissue. (A) An illustration of positive Daxx staining in GC tissue and negative staining in the adjacent normal tissue from the same patient. The rabbit polyclonal antibodies to human Daxx (1:100, California, Santa) was used used in the immunohistochemistry. The MOD of nuclear and cytoplasm was measured at 400X magnification. (B) The positive rate of nuclear Daxx staining in GC and adjacent normal tissue. In GC tissue, 75.7% (53 out of 70 cases) was positive, which was significantly higher than the positive rate of 45.7% in the adjacent normal (32 out of 70 cases, *P* < 0.001). (C) Nuclear MOD in GC and adjacent normal tissues with positive Daxx staining. No statistical difference was observed (*P* = 0.169). (D) When NCR (Nuclear/Cytoplasm Ratio of Daxx expression) was used, the GC tissue has significantly higher NCR than the adjacent normal tissue (*P* < 0.001). GC, gastric cancer; MOD, mean optical density.

**Figure 4:**
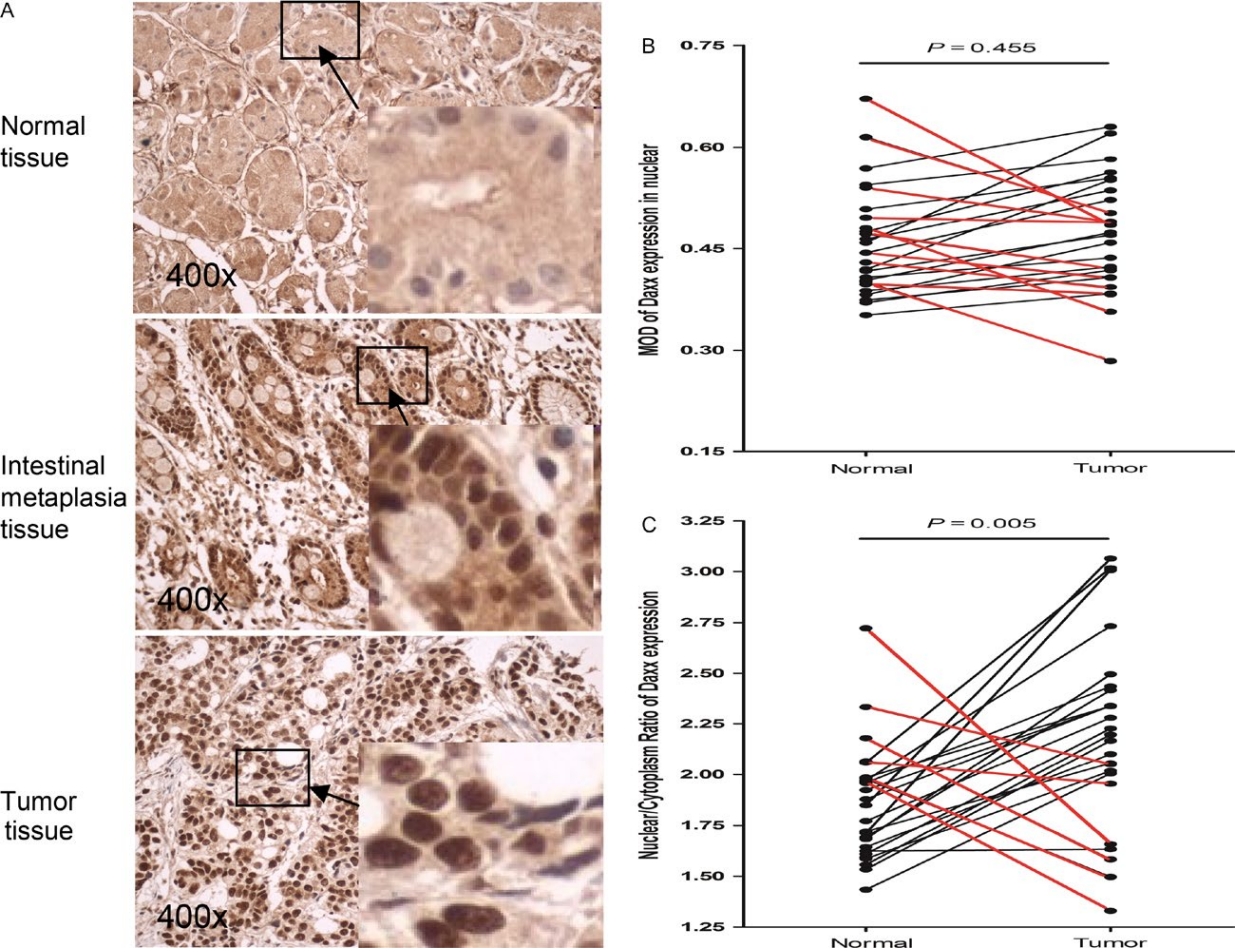
NCR of Daxx expression was more clinically informative and found higher in gastric cancer tissue than adjacent normal tissue. Rabbit polyclonal antibodies to human Daxx (1:100, California, Santa) was used in immunohistochemistry. The MOD of nuclear and cytoplasm was measured at 400X magnification. (A) Daxx expression in gastric mucosal tissue of various status. In normal gastric mucosa tissues, Daxx was found clearly expressed in the cytoplasm but not nucleus. Opposite finding was observed in gastric cancer cells. Interestingly, in the premalignant intestinal metaplasia cells, Daxx staining was found positive in both the cytoplasm and nucleus. (B) Positive nuclear MOD of Daxx expression. Using paired tumor and adjacent normal tissue, there was no statistical difference by paired *T* test (*P* = 0.455). (C) However, when using NCR (Nuclear/Cytoplasm Ratio), the Daxx NCR was found significantly higher in the tumor tissue than the matched adjacent normal tissue (*P* = 0.005). MOD, mean optical density.

Seventy pairs of matched tissues (tumor vs. adjacent normal) were analyzed by IHC, and Daxx expression was scored as positive in 53 GC tissues (75.7% of the total), but only 32 adjacent normal tissues (45.7% of the total) showed positive expression (*P* < 0.001 by paired *t*‐test) (Fig. 3B). Typical nuclear staining is shown in Figure 3A. There was no difference in nuclear MOD when GC tissue was compared with adjacent normal tissue (*P* = 0.169) (Fig. 3C). However, in Daxx‐expression positive cancerous tissue, the Daxx NCR was significantly higher than that in the tissue adjacent to the carcinoma (*P* < 0.001) (Fig. 3D). In contrast, in normal gastric mucosal cells, Daxx was clearly expressed in the cytoplasm but was absent in the nucleus, this phenomenon was detected in 38 adjacent normal tissues(70 tissues in total), also was detected in 18 normal tissues from patients who had undergone elective bariatric surgery (23 tissues in total), Interestingly, intestinal metaplasia cells appeared to express Daxx in both the nucleus and the cytoplasm, and in tumor cells, Daxx was clearly expressed in the nucleus but was absent in the cytoplasm. Typical images are shown in Figure 4A. It should be noted that, the matched tissues, which were not positive for Daxx expression in both GC and adjacent normal tissues, were eliminated from the analysis. In the remaining 26 pairs of matched GC and adjacent normal tissue samples (which were positive for Daxx expression in both tissue samples), there was no obvious difference in Daxx MOD between the GC tissue and adjacent normal tissue, *P* = 0.455 (Fig. 4B). However, an analysis of the Daxx NCR revealed that the NCR was higher in GC tissue than in adjacent normal tissue (*P* = 0.005 by paired *t*‐test) (Fig. 4C). On this basis, we conclude that there is a change in the subcellular localization of Daxx in gastric cancer, which might be associated with carcinogenesis.

### Daxx NCR ratio and the clinicopathologic data

The patients were divided into a Prior cohort (treatment between 2006 and 2009) and a Recent cohort (treatment between 2010 and 2011). Each cohort was then subdivided into a high‐risk group and a low‐risk group as mentioned above. The clinical data included gender, age, tumor size, differentiation, depth of invasion, lymph node metastasis, TNM stage, serum CEA, and serum CA 19‐9 (Table 1). In the Prior cohort, there were differences between low‐risk and high‐risk patients in terms of tumor size (*P* = 0.003), differentiation status (*P* = 0.002), depth of invasion (*P* < 0.001), lymph node metastasis (*P* < 0.001), and TNM stage (*P* < 0.001). In the Recent cohort, we observed the same phenomenon; there were significant differences between the low‐risk and high‐risk groups with respect to tumor size (*P* < 0.001), differentiation status (*P* = 0.003), depth of invasion (*P* < 0.001), lymph node metastasis (*P* < 0.001), and TNM stage (*P* < 0.001), as shown in Table 1.

**Table 1:** The clinical characteristics of patients according to the nucleoplasm ratio of DAXX in the prior and recent cohorts

Variables	Prior cohort (*n* = 277)			Recent cohort (*n* = 245)		
	Low‐risk group (*n* = 118)	High‐risk group (*n* = 159)	*P* value	Low‐risk group (*n* = 80)	High‐risk group (*n* = 165)	*P* value
Sex (*n* [%])			0.806			0.519
Female	38 (32.2%)	49 (30.8%)		26 (32.5%)	47 (28.5%)	
Male	80 (67.8%)	110 (69.2%)		54 (67.5%)	118 (71.5%)	
Age (*n* [%])			0.137			0.956
<60	70 (59.3%)	80 (50.3%)		38 (47.5%)	79 (47.9%)	
≥60	48 (40.7%)	79 (49.7%)		42 (52.5%)	86 (52.1%)	
Tumor size (cm) (*n* [%])			0.003			<0.001
<4	68 (57.6%)	63 (39.6%)		44 (55.0%)	48 (29.1%)	
≥4	50 (42.4%)	96 (60.4%)		36 (45.0%)	117 (70.9%)	
Depth of invasion (*n* [%])			<0.001			<0.001
T1 + T2	59 (50.0%)	43 (27.0%)		41 (51.2%)	38 (23.0%)	
T3 + T4	59 (50.0%)	116 (73.0%)		39 (48.8%)	127 (77.0%)	
Lymph node metastasis (*n* [%])			<0.001			<0.001
N0	63 (53.4%)	44 (27.7%)		45 (56.2%)	42 (25.5%)	
N1 + N2 + N3	55 (46.6%)	115 (72.3%)		35 (43.8%)	123 (74.5%)	
Differentiation status (*n* [%])			0.002			0.003
Well and moderate	65 (55.1%)	58 (36.5%)		46 (57.5%)	62 (37.6%)	
Poor and undifferentiated	53 (44.9%)	101 (63.5%)		34 (42.5%)	103 (62.4%)	
TNM stage (*n* [%])			<0.001^a^			<0.001^a^
Ο	8 (6.8%)	0 (0.0%)		5 (6.2%)	2 (1.2%)	
I	39 (33.1%)	24 (15.1%)		29 (36.2%)	24 (14.6%)	
II	36 (30.5%)	52 (32.7%)		18 (22.5%)	28 (17.1%)	
III	35 (29.7%)	82 (51.6%)		27 (33.8%)	105 (64.0%)	
IV	0 (0.0%)	1 (0.6%)		1 (1.2%)	5 (3.0%)	
Adjuvant chemotherapy (*n* [%])			0.083			0.058
No	41 (34.7%)	40 (25.2%)		33 (41.2%)	48 (29.1%)	
Yes	77 (65.3%)	119 (74.8%)		47 (58.8%)	117 (70.9%)	
Serum CEA (ng/mL), median (range)	2.03 (0.006–130.0)	2.12 (0.500–237.000)	0.549^a^	2.205 (0.20–92.07)	2.55 (0.010–712.0)	0.153^a^
Serum CA19‐9 (U/mL), median (range)	10.175 (0.140–1000.0)	8.75 (0.600–936.200)	0.424^a^	10.160 (0.50–415.30)	11.78 (0.41–1000.0)	0.2^a^

*χ*2 test or Fisher's exact test.

a Mann–Whitney *U* test (non‐parametric). Kruskal–Wallis rank for continuous variables and CA19‐9, carbohydrate antigen 19‐9; CEA, carcinoembryonic antigen; TNM, tumor‐node‐metastasis.

### Daxx NCR ratio and survival analysis

**Figure 5:**
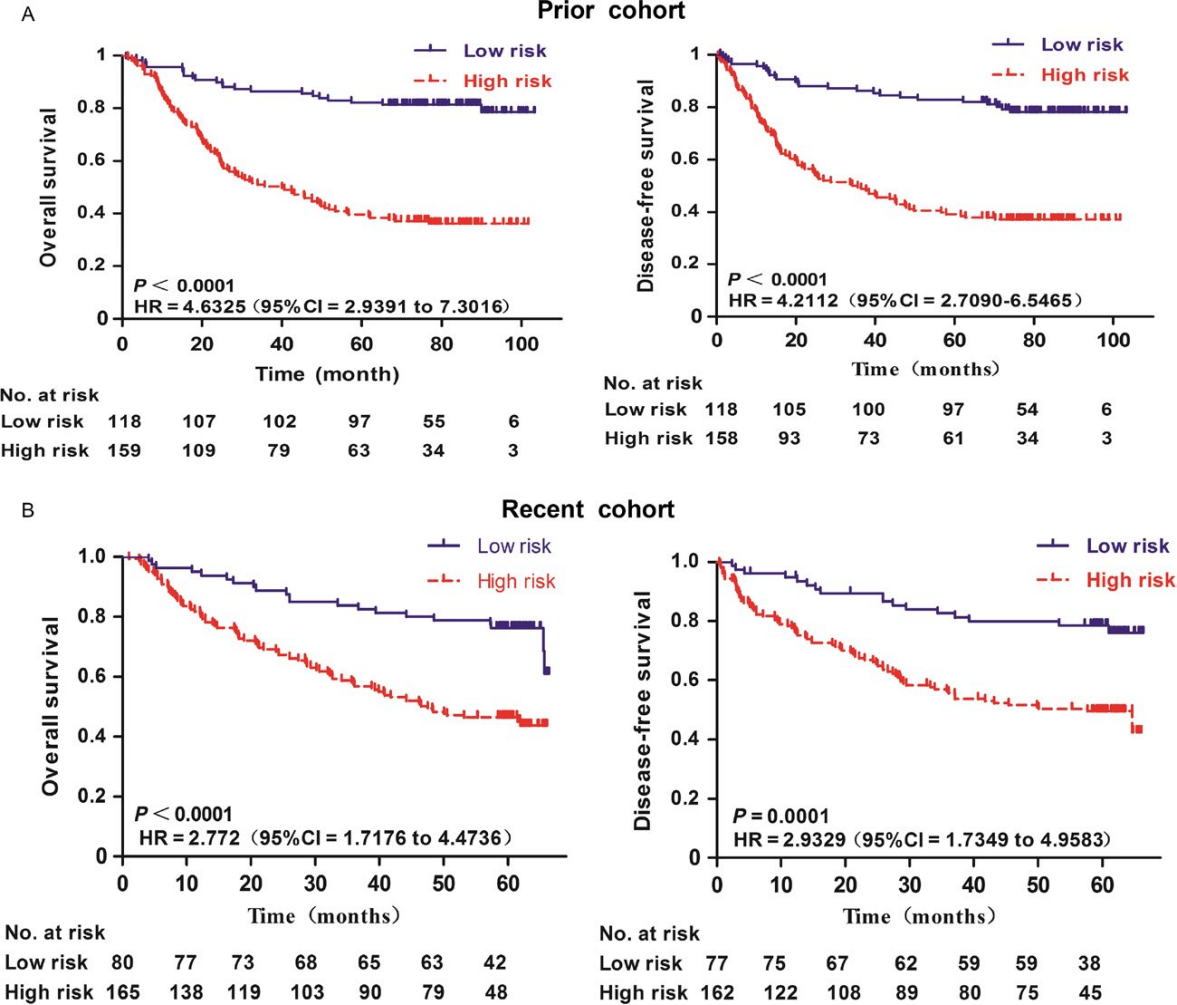
High‐risk NCR of Daxx expression was associated with poor survivals of gastric cancer patients in both the Prior and Recent cohorts. Patients were dichotomised into high and low‐risk subgroups at the cut‐off point (2.0) of the Nuclear/Cytoplasm Ratio of Daxx expression. Overall survival and Disease‐free survival are presented here. The red and blue line represents the high and low‐risk subgroup, respectively. Log‐rank *P* values are from Kaplan–Meier analysis with log‐rank test. (A) Prior cohort. (B) Recent cohort.

We conducted a survival analysis for the high‐ and low‐risk groups, and the Kaplan–Meier curve suggested that the high‐risk group had a shorter OS relative to the low‐risk group in the Prior cohort, (*P* < 0.0001, HR = 4.6325,95% CI = 2.9391–7.3016) as well as in the Recent cohort, (*P* < 0.0001, HR = 2.772,95% CI = 1.7176–4.4736). Similarly, the high‐risk group had a shorter DFS relative to the low‐risk group in both the Prior (*P* < 0.0001, HR = 4.2112,95% CI = 2.7090–6.5465) and Recent cohorts (*P* < 0.0001, HR = 2.9329,95% CI = 1.7349–4.9583) (Fig. 5).

In the Prior cohort, the result of the multivariate Cox regression analyses showed that Daxx NCR (high‐risk vs. low‐risk), tumor size (≥4 cm vs. <4 cm), and serum CEA (≥5 ng/mL vs. <5 ng/mL) were independently associated with poor DFS, whereas Daxx NCR (high‐risk vs. low‐risk), TNM stage (III + IV vs. 0 + I + II),tumor size (≥4 cm vs. <4 cm), and differentiation grade (poorly vs. well + moderately) were independent risk factors for OS (Table 2). A high‐risk Daxx NCR, rather than the other clinico‐pathological covariates, was significantly associated with unfavorable DFS and OS.

**Table 2:** Cox regression analysis of Nuclear/Cytoplasm Ratio of Daxx expression and clinicopathological covariates with survival in the Prior cohort

	Disease‐free survival				Overall survival			
	Univariate analysis		Multivariate analysis		Univariate analysis		Multivariate analysis	
	HR (95% CI)	*P* value	HR (95% CI)	*P* value	HR (95% CI)	*P* value	HR (95% CI)	*P* value
Daxx NCR (High risk vs. low risk)	4.21 (2.71, 6.55)	<0.001	2.82 (1.68, 4.73)	<0.001	4.63 (2.94, 7.30)	<0.001	2.97 (1.75, 5.02)	<0.001
TNM stage (III + IV vs. 0 + I+II)	3.79 (2.62, 5.49)	<0.001	1.78 (0.98, 3.23)	0.057	4.01 (2.76, 5.83)	<0.001	1.83 (1.01, 3.30)	0.045
Lymph nodes examined (N1 + N2 + N3 vs. N0)	3.66 (2.34, 5.72)	<0.001	1.13 (0.58, 2.19)	0.723	4.02 (2.53, 6.38)	<0.001	1.20 (0.62, 2.34)	0.591
Sex (Male vs. Female)	0.89 (0.61, 1.30)	0.556	1.01 (0.66, 1.55)	0.957	0.90 (0.62, 1.31)	0.592	1.02 (0.67, 1.56)	0.912
Age (≥60 vs. <60)	1.19 (0.84, 1.70)	0.325	1.01 (0.67, 1.53)	0.963	1.26 (0.89, 1.80)	0.195	1.09 (0.73, 1.65)	0.670
Depth of invasion (T3 + T4 vs. T1 + T2)	4.24 (2.62, 6.86)	<0.001	1.48 (0.79, 2.74)	0.218	4.46 (2.73, 7.28)	<0.001	1.42 (0.76, 2.63)	0.269
Tumor size (≥4 cm vs. <4 cm)	2.75 (1.87, 4.03)	<0.001	1.78 (1.09, 2.90)	0.021	2.87 (1.95, 4.22)	<0.001	1.76 (1.08, 2.88)	0.024
Differentiation grade (poorly vs. well + moderately)	1.72 (1.18, 2.49)	0.004	1.53 (0.99, 2.37)	0.053	1.73 (1.20, 2.51)	0.004	1.61 (1.05, 2.49)	0.030
Adjuvant chemotherapy (yes vs. No)	2.08 (1.32, 3.26)	0.002	1.83 (1.02, 3.28)	0.042	1.88 (1.21, 2.92)	0.005	1.63 (0.93, 2.84)	0.086
Serum CEA (ng/mL) (≥5 vs. <5)	1.54 (0.98, 2.41)	0.063	1.25 (0.78, 2.02)	0.357	1.68 (1.09, 2.61)	0.020	1.38 (0.87, 2.19)	0.175
Serum CA19‐9 (U/mL) (≥37 vs. <37)	1.50 (0.85, 2.64)	0.159	1.29 (0.72, 2.32)	0.396	1.50 (0.85, 2.63)	0.158	1.31 (0.73, 2.36)	0.370

Daxx NCR, Nuclear/Cytoplasm Ratio of Daxx expression; CA19‐9, carbohydrate antigen 19‐9; CEA, carcinoembryonic antigen; TNM, tumor to node to metastasis.

In the Recent cohort, Daxx NCR (high‐risk vs. low‐risk), lymph nodes examined (N1 + N2 + N3 vs. N0), and depth of invasion (T3 + T4 vs. T1 + T2) were independently associated with poor DFS, while Daxx NCR (high‐risk vs. low‐risk), lymph nodes examined (N1 + N2 + N3 vs. N0), depth of invasion (T3 + T4 vs. T1 + T2), and tumor size (≥4 cm vs. <4 cm) were independent risk factors for OS (Table 3).

**Table 3:** Cox regression analysis of Nuclear/Cytoplasm Ratio of Daxx expression and clinicopathological covariates with survival in the Recent cohort

	Disease‐free survival				Overall survival			
	Univariate analysis		Multivariate analysis		Univariate analysis		Multivariate analysis	
	HR (95% CI)	*P* value	HR (95% CI)	*P* value	HR (95% CI)	*P* value	HR (95% CI)	*P* value
DaxxNCR (High risk vs. low risk)	2.93 (1.73, 4.96)	<0.001	1.93 (1.10, 3.39)	0.023	2.77 (1.72, 4.47)	<0.001	1.90 (1.12, 3.21)	0.017
TNM stage (III + IV vs. 0 + I + II)	6.80 (3.85, 12.01)	<0.001	1.11 (0.47, 2.61)	0.813	7.22 (4.24, 12.30)	<0.001	1.16 (0.51, 2.62)	0.725
Lymph nodes examined (N1 + N2 + N3 vs. N0)	10.26 (4.75, 22.17)	<0.001	4.18 (1.45, 12.00)	0.008	9.65 (4.87, 19.11)	<0.001	3.21 (1.18, 8.69)	0.022
Sex (Male vs. Female)	1.17 (0.74, 1.84)	0.506	0.75 (0.44, 1.29)	0.302	1.24 (0.81, 1.89)	0.329	0.77 (0.47, 1.28)	0.315
Age (≥60 vs. <60)	0.99 (0.67, 1.48)	0.974	1.05 (0.67, 1.64)	0.842	1.23 (0.81, 1.79)	0.279	1.26 (0.82, 1.92)	0.291
Depth of invasion (T3 + T4 vs. T1 + T2)	7.91 (3.83, 16.33)	<0.001	2.60 (1.06, 6.35)	0.037	10.52 (4.89, 22.65)	<0.001	3.28 (1.34, 8.01)	0.009
Tumor size (≥4 cm vs. <4 cm)	4.31 (2.48, 7.48)	<0.001	1.73 (0.93, 3.24)	0.082	5.13 (3.01, 8.72)	<0.001	2.22 (1.20, 4.08)	0.011
Differentiation grade (poorly vs. well + moderately)	1.22 (0.82, 1.83)	0.333	0.84 (0.51, 1.36)	0.468	1.08 (0.74, 1.57)	0.685	0.69 (0.44, 1.10)	0.120
Adjuvant chemotherapy (yes vs No)	2.96 (1.75, 5.00)	<0.001	1.12 (0.62, 2.00)		3.02 (1.82, 4.95)	<0.001	1.24 (0.71, 2.14)	0.450
Serum CEA (ng/mL) (≥5 vs. <5)	2.12 (1.38, 3.25)	<0.001	1.07 (0.63, 1.83)	0.711	2.28 (1.53, 3.41)	<0.001	0.94 (0.57, 1.56)	0.816
Serum CA19‐9 (U/mL) (≥37 vs. <37)	1.81 (1.16, 2.82)	0.009	0.96 (0.58, 1.58)	0.857	2.10 (1.40, 3.15)	<0.001	1.10 (0.69, 1.74)	0.696

Daxx NCR, Nuclear/Cytoplasm Ratio of Daxx expression; CA19‐9, carbohydrate antigen 19‐9; CEA, carcinoembryonic antigen; TNM, tumor to node to metastasis.

### Daxx NCR ratio and postoperative adjuvant chemotherapy

**Figure 6:**
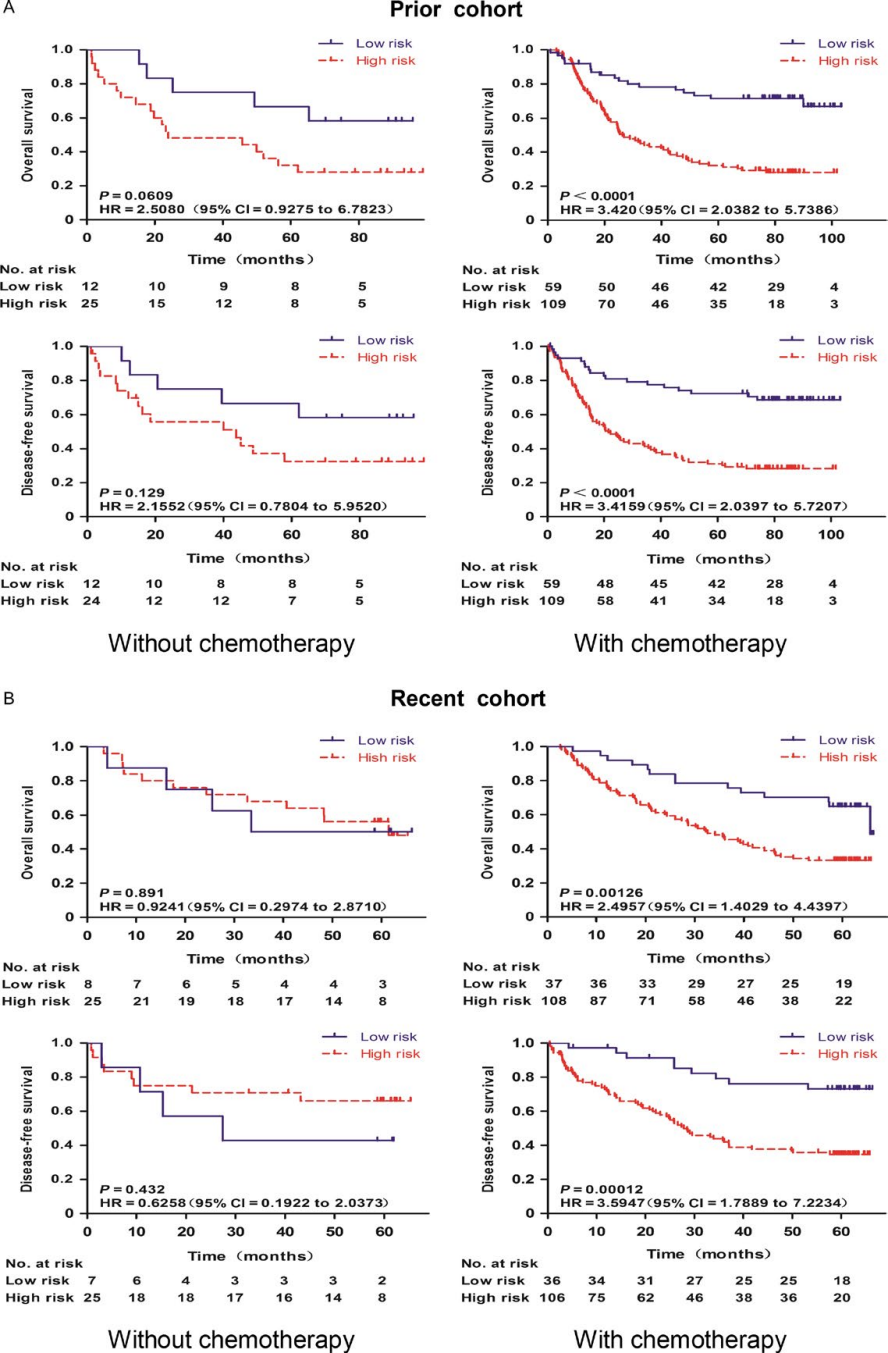
The effect of postoperative chemotherapy on the survivals of high versus low‐risk patients with stage II + III gastric cancer in both the Prior and Recent cohorts. Both the overall suvival and disease‐free survival were analyzed. In either cohort, the patients with stage II + III gastric cancer were dichotomised into high‐risk subgroup and low‐risk subgroup at the cut‐off point (2.0) of the Nuclear/Cytoplasm Ratio. Overall survival and Disease‐free survival of the patients with and without chemotherapy are presented. *P* Values are shown. The red and blue line represents the high and low‐risk subgroup, respectively. Log‐rank *P* values are from Kaplan–Meier analysis with log‐rank test. (A) Prior cohort. (B) Recent cohort.

We also divided the patients with stage II and III gastric cancer into two groups, either with or without adjuvant chemotherapy, and asked if Daxx NCR could predict the benefit from adjuvant chemotherapy. Interestingly, in both the Prior and Recent cohorts, for patients who had received postoperative adjuvant chemotherapy, the high‐risk group was found to have a shorter DFS compared to the low‐risk group (Fig. 6, Prior cohort: *P* < 0.0001, HR = 3.4159,95% CI = 2.0397–5.7207; Recent cohort: *P* = 0.00012, HR = 3.5947,95% CI = 1.7889–7.2234). Such a difference in DFS was not observed between the high‐ and low‐risk patients who did not receive adjuvant chemotherapy. Similar result was observed for OS; the high‐risk group had a shorter OS in both the Prior (*P* < 0.0001, HR = 3.420,95% CI = 2.0382–5.7386) and Recent cohorts (*P* = 0.00126, HR = 2.4957, 95% CI = 1.4029–4.4397) in patients who received adjuvant chemotherapy. However, for those who had no adjuvant chemotherapy, no statistical difference was observed (Fig. 6). These findings suggest that adjuvant chemotherapy has a differential effect on high‐ and low‐risk patients. To better characterize this effect, we pooled the Prior and Recent cohorts to increase the sample size. As shown in FigureS2, postoperative chemotherapy clearly provided no benefit to the high‐risk patients with elevated Daxx NCR. In fact, we observed the trend of worsening DFS in high‐risk patients who received adjuvant chemotherapy (*P* = 0.062). Future studies, with an increased sample size, are warranted to confirm this finding.

## Discussion

In this study, Daxx expression in tumor cells was found to be higher than that in adjacent normal gastric mucosa cells (Fig. 3). Expression of Daxx has been studied in a variety of tumors 8, 9, 11, 12, 14, 20, 21, 22. However, all the reported studies have been limited in that they were performed in vitro and in tumors other than gastric cancer. The question of whether the expression or localization of Daxx is related to the aggressiveness of gastric carcinoma has not been addressed. Historically, gastric cancer research has been guided by prior studies conducted in other cancer types. For example, one report examined the potential mechanism of the breast cancer gene Her‐2 in gastric carcinoma 23. However, research on gastric cancer is now progressing along its own pathway, and with the development of gastric carcinoma‐related gene sequencing technology, more and more potentially important genes such as FAT4 and ARID1A are being discovered 24, 25, with more being reported recently 26, 27. However, although many genes have now been reported in gastric cancer studies, the role of Daxx has yet to be identified. This report is by far the first to investigate the relationship between Daxx and the prognosis of gastric carcinoma.

Comparing nuclear expression in Daxx‐positive cancer tissues with that in adjacent Daxx‐positive normal tissues revealed no significant difference between the two tissue types. However, a comparison of the Daxx nuclear/cytoplasmic ratio revealed that this ratio was significantly higher in cancer tissues than in adjacent normal tissues (Fig. 4). Previous studies have not made a connection between the Daxx nuclear/cytoplasmic expression ratio and GC, and this is the first study of its type to use Daxx IHC in clinical gastric carcinoma tissue. Numerous previous studies 15, 16, 17, 18, 19 have shown that protein subcellular localization can be closely related to tumor progression. In this study, we found that the subcellular localization of Daxx in cells is complex, because it is expressed in both the nucleus and the cytoplasm. Our study found that the expression of Daxx subcellular localization differed between GC tissues and adjacent normal tissues. In tumor cells, Daxx expression in the nucleus was significantly higher than that in the cytoplasm, but in normal gastric mucosa cells, exactly the opposite was observed, with Daxx being found primarily in the cytoplasm. Interestingly, in the intestinal metaplasia (considered as premalignant lesion) of the gastric mucosa, Daxx was found having its expression in both the cytoplasm and nucleus (Fig. 4). Since cancer can take a long time to develop, this phenomenon may, in the future, provide us with a new means of early diagnosis of gastric cancer or screening of high‐risk population for gastric cancer. The relationship between nuclear Daxx accumulation and the mechanism by which mucosal cells become cancerous is unclear. It is possible that this may reflect a new mechanism that could help us understand the development of gastric carcinoma, and in the future, we plan to perform more in depth studies to address this.

In the Prior cohort, the NCR was found to be closely related to the poorer prognosis of gastric cancer. This phenomenon was also confirmed in the Recent cohort (Table 1). Interestingly, our analysis revealed that Daxx NCR is related to OS and DFS in patients; compared to the low‐risk group, the high‐risk group presented with shorter DFS and OS (Fig. 5). This study therefore suggests that nuclear Daxx might have a role in promoting tumor progression. This study is also the first to show the potential association of Daxx distribution with the clinical pathological characteristics of gastric cancer and use this to evaluate the prognosis of gastric cancer patients.

In patients with advanced gastric cancer, in order to better control the recurrence rate after surgery, various adjuvant therapies have been employed. Postoperative adjuvant chemotherapy, for example, has been shown to improve the survival rate of patients with gastric cancer after radical surgery 28, 29, 30, 31, 32. However, chemotherapy is not without adverse effects. Predicting the benefit from chemotherapy is therefore clinically important, especially for patients who have already undergone radical gastrectomy. Our study clearly demonstrated that postoperative chemotherapy has a differential effect on high versus low‐risk patients (stage II and III GC) based on the Daxx NCR. While chemotherapy did not bring any survival benefit for high‐risk patients after surgery, our data suggested it could even be detrimental based on the worsening trend in DFS, although a prospective study with a bigger sample size is needed to confirm. Despite a recent study which suggested that high microsatellite instability and mismatch repair deficiency were negatively correlated with survival in gastric cancer patients who underwent perioperative chemotherapy [33], there is so far no definitive biomarker to guide us in selecting the right population for perioperative and/or adjuvant chemotherapy. The Daxx NCR could therefore be clinically valuable that is worth further investigation.

In summary, we found that enhanced nuclear accumulation of Daxx correlated with the malignant phenotype in gastric mucosa. Further mechanistic studies will be needed to understand the relationship between nuclear accumulation of Daxx and gastric cancer. In addition, through the innovative use of the Daxx NCR, for the first time, we have clearly demonstrated that elevated Daxx NCR is associated with a poorer clinical prognosis in gastric cancer patients. More importantly, we observed no benefit (and could be potentially detrimental) from postoperative chemotherapy for patients with elevated Daxx NCR, with a suggestion that it could in fact be detrimental, suggesting that Daxx NCR may be valuable in clinical decision‐making that may warrant a prospective study to confirm.

## Conflict of Interest

None declared.

## Supporting information


**Figure S1.** Immunohistochemical results of GC tissues and adjacent normal tissues.

**Figure S2.** The effect of postoperative chemotherapy on high versus low‐risk patients (stage II + III gastric cancer) based on Daxx NCR.
