# Supplementary figures and images for: Prognostic significance of Daxx NCR (Nuclear/Cytoplasmic Ratio) in gastric cancer
Source: Cancer Med. 2017 Aug 15;6(9):2063–75. doi: 10.1002/cam4.1144 (PMC5603835; doi:10.1002/cam4.1144)

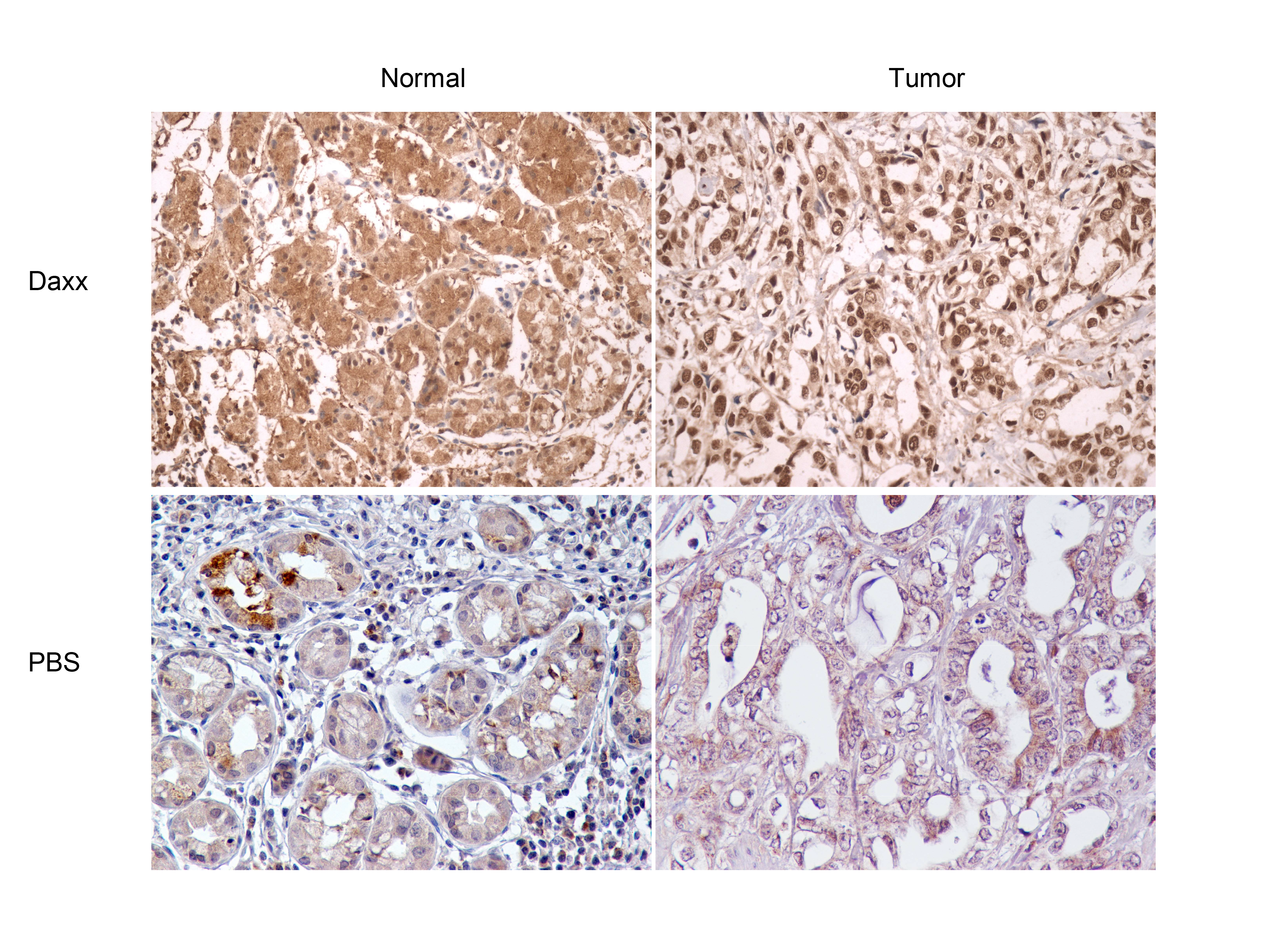

Supplement: Supplementary file 1 — Figure S1. Immunohistochemical results of GC tissues and adjacent normal tissues. [file CAM4-6-2063-s001.tif]

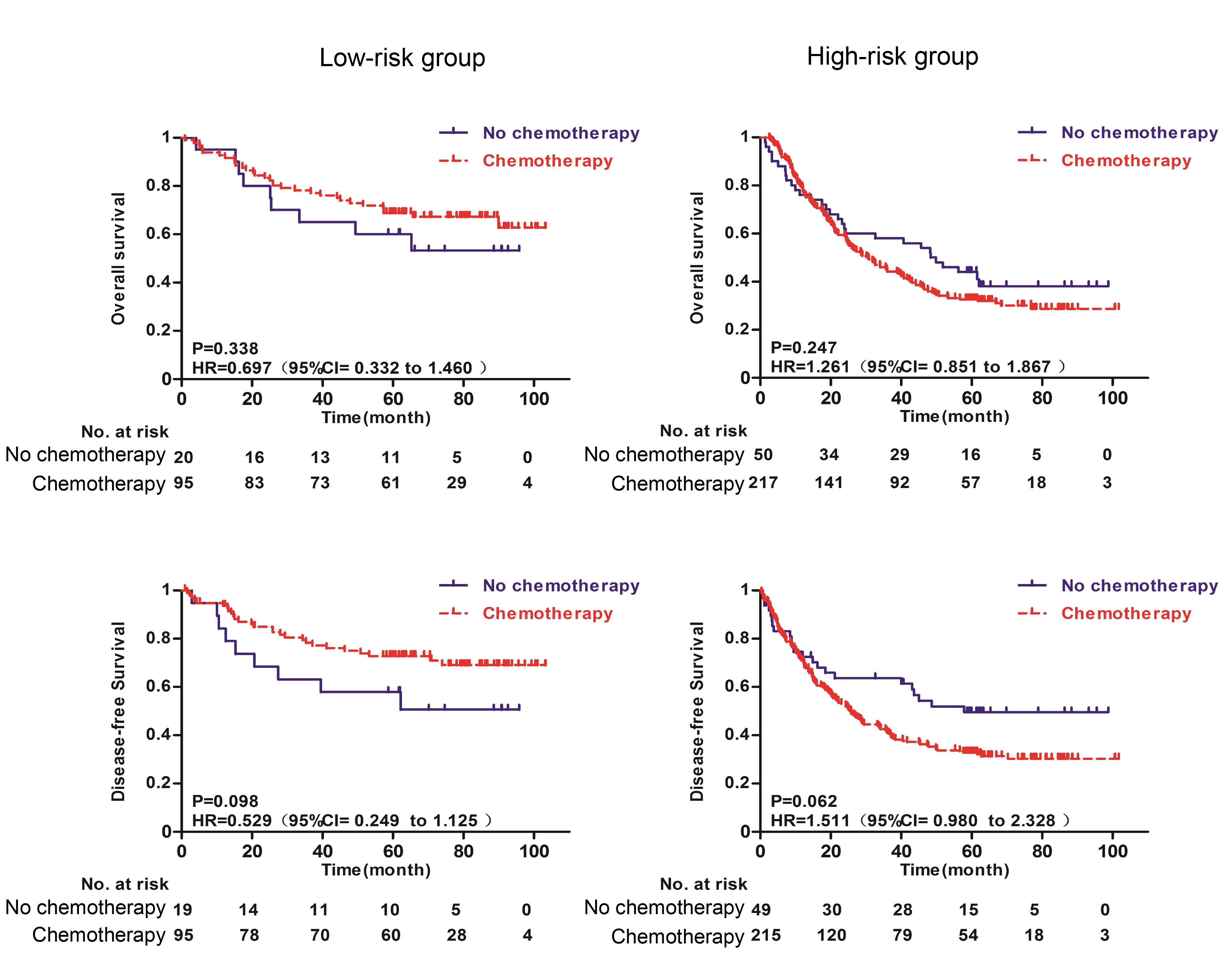

Supplement: Supplementary file 2 — Figure S2. The effect of postoperative chemotherapy on high versus low‐risk patients (stage II + III gastric cancer) based on Daxx NCR. [file CAM4-6-2063-s002.tif]
